# Direct-Acting Antivirals Are a Milestone for Hepatitis C Virus Infection? Analysis of 15 Years of Patient and Diagnosis Data from a Region in Türkiye

**DOI:** 10.3390/jcm15072678

**Published:** 2026-04-01

**Authors:** Yusuf Yakupogullari, Elif Seren Tanrıverdi, Baris Otlu

**Affiliations:** Department of Medical Microbiology, Faculty of Medicine, Inonu University, 44280 Malatya, Türkiye; seren.tanriverdi@inonu.edu.tr (E.S.T.); botlu@yahoo.com (B.O.)

**Keywords:** hepatitis C, DAA, epidemiology, elimination, pandemic, earthquake

## Abstract

**Background:** The landscape of hepatitis C virus (HCV) infection has been changing with the introduction of direct-acting antivirals (DAAs). This study evaluates 15-year temporal trends of anti-HCV and HCV-RNA positivity in a regional referral center in Türkiye, analyzing the impact of DAA treatments, the COVID-19 pandemic, and the 2023 earthquakes on disease dynamics. **Methods**: Laboratory data of patients tested for anti-HCV antibodies and HCV-RNA between 2011 and 2025 were retrospectively analyzed after excluding repeat records. Positive patients were categorized by antibody titers (1–4.99 S/Co and ≥5 S/Co) and viremia status. Poisson, beta, and quantile regression models were determined annual trends in case numbers, positivity rates, and median ages. **Results**: A total of 402,557 patients underwent anti-HCV screening over 15 years. While annual test volume increased 2.25-fold, the number and rate of high-titer (≥5 S/Co) positive patients decreased four-fold, significantly. HCV-RNA positivity rates remained stable between 2011 and 2016 but declined sharply from 2017, falling approximately 19.2% annually (*p* < 0.001). Significant diagnostic disruptions occurred in 2020 (pandemic) and 2023 (earthquakes). An “aging trend” was identified; the median age of viremic patients increased by over 5.5 years throughout the study period. **Conclusions**: The introduction of DAAs in 2016 marked a milestone, leading to a nearly 90% reduction in the viremic patient burden in our region. The steady aging of the HCV-positive population suggests that the infected pool is shrinking and is not replenished. However, global and regional crises can hinder screening efforts, necessitating resilient public health strategies to achieve World Health Organization 2030 elimination targets.

## 1. Introduction

Hepatitis C virus (HCV) is a hepatotropic virus belonging to the Flaviviridae family with a 50–60 nm enveloped virion structure carrying (+) polarity ssRNA genome and is one of the most important causes of chronic viral hepatitis worldwide [1]. Although it varies depending on age of acquisition, chronic progression in infected individuals is as high as 60–80% [2]; and it is estimated that at least 20% of global hepatocellular carcinoma (HCC) cases [3] and more than 25% of cirrhosis cases are caused by HCV [4]. According to World Health Organization (WHO) data, as of 2022, approximately 50 million people in the world are living with chronic HCV and about 240 thousand people die annually as a result of HCV-related liver diseases [1]. Analyses show that Türkiye is a low-prevalence country for HCV infection as anti-HCV positivity rate in Turkey is around 0.3–1%, and the prevalence of active infection (HCV RNA positive) is around 0.3% [5,6].

Transmission of HCV occurs mainly through blood routes such as unsafe blood transfusions and dialysis. The majority of acute infections in the pediatric population occur perinatally, with a 5–8% transmission rate from infected mother to infant [7]. The other common route of transmission in adults includes unsafe medical practices such as unsterile injections, dental procedures and other minor surgical interventions performed under non-sterile conditions. Additionally, tattooing and piercing with contaminated tools, shared drug injectors and sexual intercourse cause tens of thousands of new HCV infections every year worldwide [8].

In 2016, WHO announced a health sector strategy to reduce the global public health burden of HCV, which includes reducing new chronic HCV infections by 80% and HCV-related deaths by 65% by 2030; ensuring that at least 90% of cases are diagnosed and at least 80% of those diagnosed have access to treatment [9].

The introduction of direct-acting antivirals (DAAs) has fundamentally transformed the global strategy for hepatitis C elimination. With sustained virological response rates exceeding 95%, DAAs not only improve individual patient outcomes but also significantly reduce viral transmission at the population level. Therefore, the scale-up of DAA-based treatment programs has become the cornerstone of the World Health Organization’s goal to eliminate hepatitis C as a public health threat by 2030. Türkiye published two prevention and control programs, the first in 2017 and the second in 2025 [6] and announced action policies under the following main headings: increasing awareness and immunization against viral hepatitis, preventing mother-to-infant transmission, providing safe blood and blood products, preventing transmission in intravenous drug users and prisoners, and preventing healthcare-related transmission. In addition, laws have been enacted in Türkiye to eliminate unsafe health practices and intensive inspections have been initiated. A blood test for HCV was required for marriage applicants, and if one spouse tested positive, information and counseling actions were implemented to prevent transmission to the other spouse. Pregnant women were included in the scope of routine screening for HCV and measures were implemented to prevent transmission to the baby. According to the recommendation of The Turkish Association for the Fight Against Viral Hepatitis, screening is advised for the spouse/sexual partners and household contacts of HCV-positive individuals sharing personal items. DAAs, which achieve sustained virological response (SVR) rates exceeding 95% [10], have been provided free of charge under general health insurance as of May 2016 in the country, and the HCV patients who were followed until that date were called for the treatment initiation. Finally, modeling studies have reported predictions that preventive and therapeutic practices that have been carried out for nearly 20 years will have positive results in reducing the HCV burden of our country [11]. Additionally, it was reported that anti-HCV antibody positivity in intravenous drug users decreased by nearly 8% from 2019 to 2024 in the report published by the National Department of Combating Narcotic Crimes [12]. However, according to the best of our knowledge, there is still no study that analyzes long-term data on how laboratory-based HCV positivity trends have changed despite all the above-mentioned measures that have intensified in the last two decades. Whereas such analyses provide critical data for determining the best action and improving the effectiveness of preventive and therapeutic interventions.

This study aimed to determine changes in HCV test results and the demographic characteristics of the tested patients over the last 15 years at a tertiary-level hospital serving as a referral center for gastroenterology and hepatology in a large geographical area.

## 2. Materials and Methods

### 2.1. Study Design and Frame

This study was conducted at the İnönü University Turgut Ozal Medical Center (TOMC), a 1500-bed tertiary regional reference hospital. As the leading facility in Turkey for liver transplantation capacity, the center serves as a regional hub for gastroenterology and hepatology healthcare, supported by specialized adult and pediatric gastroenterology and infectious diseases clinics.

Since early 2000s, TOMC has functioned as the primary referral point for all healthcare facilities in the region. Patients with suspected HCV infection or those testing anti-HCV positive are routinely referred here for comprehensive serological and molecular diagnostic confirmation, as well as for the management of their subsequent treatment processes.

The study was carried out under the approval from the İnönü University Health Sciences Scientific Research Ethics Committee (Approval No: 2026/9217; Approval Date: 13 January 2026).

### 2.2. Data Collection and Calculations

We collected data from anti-HCV antibody tests and HCV-RNA PCR assays performed in the Microbiology and Molecular Microbiology laboratories between 1 January 2011, and 31 December 2025. Demographic information, such as age and gender, alongside laboratory results, was systematically extracted from the hospital’s laboratory information management system (LIMS). Annual patient lists were generated for positive and negative cases detected between January 1 and December 31 of each year. The repeat records were excluded from those lists and annual positivity rates, and case numbers were calculated accordingly.

All laboratory data were retrieved from the same institutional LIMS. Although minor updates in laboratory infrastructure may have occurred over time, comparable assay platforms and consistent testing principles were maintained throughout the study period to ensure data reliability and comparability.

To ensure the accuracy of the annual positivity rate and incident case calculations, we applied a filtering protocol for repeat testing within each calendar year. For patients who underwent the same test multiple times in a year, only one representative record was included. For anti-HCV antibody analysis, we kept the highest result of the year and excluded the repeat entries. For HCV-RNA PCR tests, only the highest result from each year was included and repeat entries were excluded.

To determine incident cases, we first generated a list of positive patients identified within each calendar year and then produced a master list including the records of all study years. We retained only the first positive record for each individual and systematically purged all subsequent repeat entries. Each patient was then permanently assigned to the year of their initial diagnosis. To prevent the 2011 baseline data from being distorted by pre-existing cases, we cross-referenced our findings with positive patient records from previous years to ensure that ‘newly added’ patients were not simply carry-overs from the recent past.

### 2.3. Laboratory Assays: Anti-HCV and HCV-RNA PCR

Serum or plasma samples were analyzed for anti-HCV antibodies via chemiluminescent microparticle immunoassay using the Alinity kits in Alinity-i macro-ELISA platform (Abbott Laboratories, Abbott Park, IL, USA). Results were reported as the ratio of the sample relative light unit (RLU) to the cutoff RLU (S/Co). Following the manufacturer’s guidelines, a S/Co ratio ≥1.0 was considered positive. We stratified positive results into three groups: 1-overall positivity (≥1 S/Co), 2-low-titer positives (1.0–4.99 S/Co) and 3-high-titer positives (≥5.0 S/Co).

Qualitative and quantitative analysis of HCV-RNA in patients’ blood samples were studied through real-time PCR method, using artus HCV QS-RGQ V1. kits on the RotorGene-Q RT-qPCR device (Qiagen, Hilden, Germany). We interpreted measurements using internal, negative, and positive controls.

### 2.4. Statistical Analysis

We analyzed temporal changes in patient age (median; min-max), annual test volumes, and the rates of positivity using specific regression models to account for the nature of the data. Poisson regression was used for count-based (numerical) data, while beta regression was employed for proportional and rate-based data. Additionally, quantile regression was used to evaluate shifts in median age over the years. A *p*-value of <0.05 was accepted as statistical significance.

Model assumptions were evaluated prior to analysis. For Poisson regression, overdispersion was assessed and model fit was checked using residual diagnostics. Beta regression models were applied to proportional data bounded between 0 and 1, and goodness-of-fit was evaluated. Quantile regression was used to assess median trends without assuming normal distribution. All analyses were performed using R software (version 4.4.1), and results were reported with corresponding coefficients (β), standard errors (SE), and *p*-values.

## 3. Results

We analyzed anti-HCV antibody results of a total of 402,557 patients, comprising 210,339 (52.3%) males and 192,218 (47.7%) females. Out of this cohort, 7310 (1.82%) patients tested positive (≥1 S/Co). Among those with positive results, 3917 were male and 3393 were female; notably, 4177 (63.6%) of these individuals exhibited high antibody titers (≥5 S/Co). Annual distributions of test volumes, results, and demographic characteristics are presented in Table 1.

**Figure 1:**
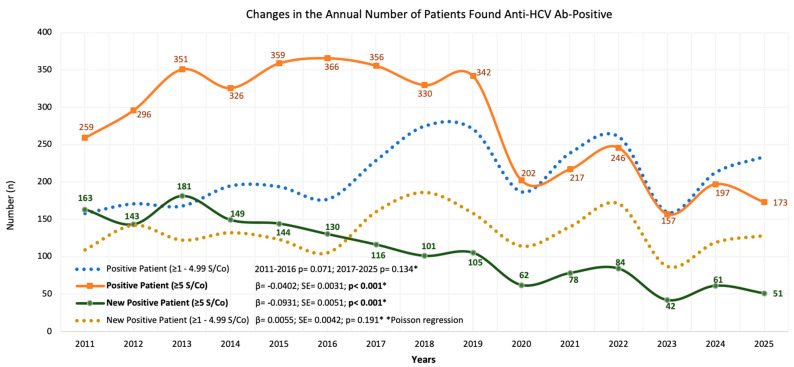
Annual trend analysis of the change in the number of patients with positive anti-HCV antibody test results. Positive patients were divided into two groups based on antibody titer (those with antibody titer ≥1–4.99 S/Co and those with antibody titer ≥5 S/Co) and into two groups: newly diagnosed and total positives. Poisson regression analysis showed that the number of positive patients with high tires of anti-HCV antibody decreased significantly by the time. While the number of positive patients with ≥5 S/Co antibody titer (high probability for true HCV infection) was apparently higher than those who were ≥1–4.99 S/Co positive, the situation reversed after 2020. A similar picture holds for the annual data on newly diagnosed patients, with a significant decrease in the number of newly diagnosed high titer (≥5 S/Co) positive patients after 2016. Both the number of new and total ≥1–4.99 S/Co positives showed insignificant fluctuations during the study period.

The number of patients tested annually increased by 2.25-fold during the study period, but the number of positive cases followed a fluctuating but ultimately downward trend. The overall positivity rate declined approximately 2.5-fold, and Poisson regression analysis revealed that there was a significant annual decrease in the number of patients with high-titer (≥5 S/Co) anti-HCV results. Conversely, the number of patients with low-titer results (≥1.0–4.99 S/Co)—which was considered a weaker diagnostic criterion for active HCV infection—showed no statistically significant change. The annual shift in anti-HCV positive patient counts is illustrated in Figure 1.

**Figure 2:**
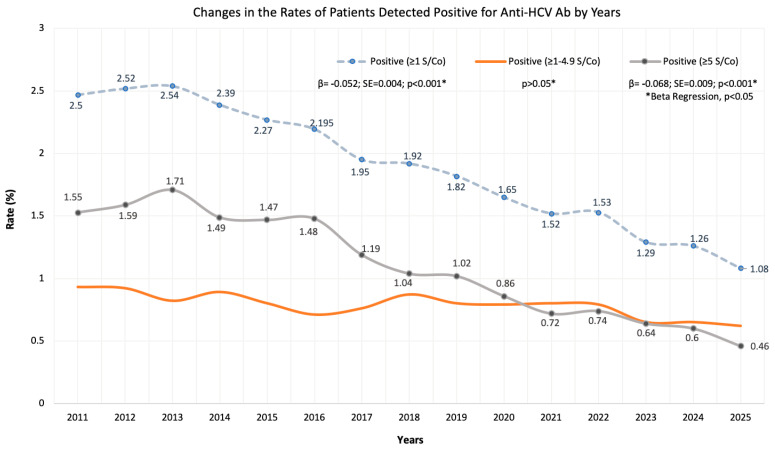
Rate of anti-HCV positivity among patients tested between 2011 and 2025. Positive cases were categorized into three groups: ‘all positives’ (≥1 S/Co), ‘positive with intermediate antibody titer’ (≥1–4.99 S/Co) and ‘high-titer’ positives (≥5 S/Co). Beta regression analysis revealed statistically significant downward trends in the rates of ‘all positives’ and ‘high-titer’ positives throughout the study period, while the rate of patients with low-titer positivity (1–4.99 S/Co) fluctuated. However, the total number of positive cases did not change significantly; these downward trends were caused by a significant increase more than twofold (β = 0.048, SE = 0.002, *p* < 0.001; Poisson regression) in the annual number of tests performed. Although there was a slight, non-significant upward trend in high-titer cases between 2011 and 2016 (β = 0.012, SE = 0.015, *p* = 0.45; Poisson regression), the substantial decline observed in the nine years following 2016 (β = −0.058, SE = 0.01, *p* < 0.001; Poisson regression analysis) resulted in a significant downward trend across the entire 15-year period. Annual test volumes are presented in Table 1.

Beta regression analysis confirmed significant declines in the proportions of both total anti-HCV positive and high-titer (≥5 S/Co) cases, with reductions of 2.5 and 4-fold, respectively (Figure 2).

**Figure 3:**
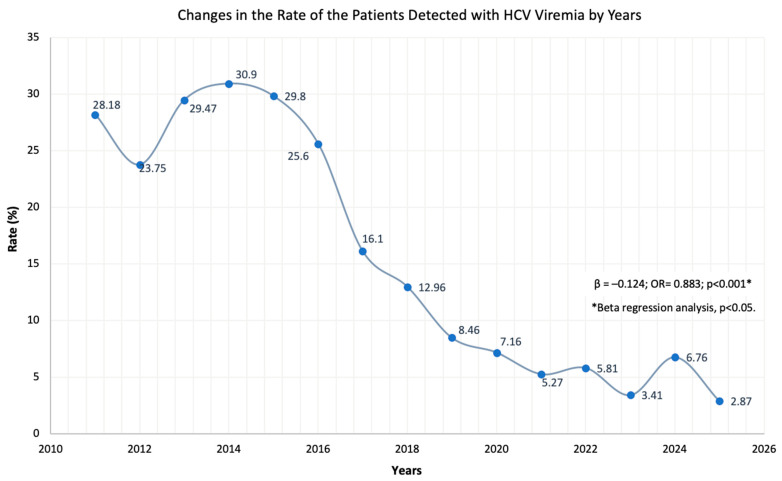
Annual changes in the rate of positive HCV-RNA-PCR results over time. Beta regression analysis revealed a statistically significant downward trend throughout the study period. This parameter was also analyzed in two periods: 2011–2016 and 2017–2025. Although there was no significant change in the 2011–2016 period (β = −0.015, SE = 0.018, *p* = 0.354), a strong downward trend was detected in the 2017–2025 period (β = −0.192, SE = 0.021, *p* < 0.001), indicating a fall of approximately 17.5% each year after 2017.

A total of 11,329 HCV-RNA PCR assays were performed for 8094 patients, and HCV viremia confirmed in 1416 individuals (17.5%). We observed a sharp decline in testing volumes: the number of patients undergoing HCV-RNA testing dropped by approximately 55% starting in 2020, while the count of HCV-RNA positive patients decreased by roughly 40% from 2017 onwards. Data for HCV-RNA PCR testing, including patient demographics, are shown in Table 2, and annual decrease in the rate of patients with detectable HCV viremia is shown in Figure 3.

**Figure 4:**
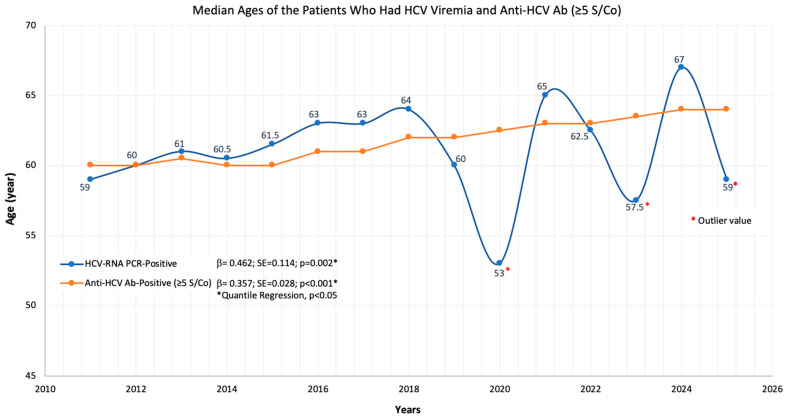
Annual change in the median age of patients with positive HCV-RNA PCR and anti-HCV antibody (≥5 S/Co) test results. Quantile regression analysis showed that the median age of patients testing positive for anti-HCV antibodies or having HCV viremia increased by more than five years throughout the study period. Data from 2020, 2023 and 2025 were considered outliers (for HCV viremia) and excluded from the calculation due to the very low number of patients in these years.

The median age of patients with high-titer anti-HCV (≥5 S/Co) results increased by four years, while the median age of those with confirmed HCV-RNA positivity rose steadily by seven years. Quantile regression analysis confirmed that both increases were statistically significant (see Figure 4), indicating an aging trend within the chronically infected population.

## 4. Discussion

In this study, we analyzed 15 years of results from a tertiary level hospital serving a region of approximately one million people and providing healthcare services to an average of 750,000 patients per year. We examined changes in two important HCV-related indicators, anti-HCV antibody positivity and HCV-RNA positivity, over the years, revealing significant decreases in these parameters over time. The 15-year scope of the study enables us to analyze the potential impact of significant events such as the introduction of effective antivirals developed against HCV during this period, the emergence of the SARS-CoV-2 virus in 2020 and the major earthquakes that occurred in our region in 2023, on laboratory-based HCV positivity trends in a given area. Analysis of the available scientific data showed that no such comprehensive study has previously been reported from our country, and few such studies exist internationally. Our study provides a valuable model for measuring changes in laboratory-based HCV positivity and viremia trends in a regional referral setting.

Anti-HCV antibody screening is the first-line diagnostic test for HCV infection, but a positive (≥1 S/Co) result does not always indicate a true infection. Studies have shown that only 7.9–23.8% of people with a positive test result of 1–4.99 S/Co are actually infected with HCV, and an anti-HCV antibody level of ≥5 is associated with a sensitivity, specificity, negative and positive predictive value of 92–99% in the diagnosis of HCV infection [13,14,15]. In our study, we analyzed anti-HCV-antibody-positive patients by dividing them into two groups: patients with an antibody titer of 1–4.99 S/Co (low titer positives) and ≥5 S/Co, and we accepted ≥5 S/Co as a reliable cut-off value for true HCV infection. It should be emphasized that antibody titers are not intended to replace confirmatory molecular testing. HCV-RNA detection remains the gold standard for diagnosing active infection and should be performed regardless of S/Co values in clinical practice. Additionally, the 1–4.99 S/Co range in anti-HCV testing may be influenced by the technical limitations of the assay and should therefore be interpreted with caution.

Our data show that the number of patients having anti-HCV antibody test has steadily increased over the years, about 2.25-fold in total. On the other hand, the decline in the number and rate of positive patients detected suggests that the increase in the number of antibody tests is not due to a spreading infection, but to an increase in awareness of HCV among the public and medical professionals. Especially after the widespread use of DAAs, such awareness raising has become even more important for the eradication of the disease. In their guidelines and recommendations, the Ministry of Health and the World Health Organization have determined the identification of individuals who are unaware of their disease in the community, providing access to the medical care system, and raising awareness about treatment and other measures to reduce infectiousness as one of the main goals [6,9]. Therefore, the increased amount of testing detected in our study was a positive finding in terms of meeting such a goal set by health authorities.

In our study, the number of ≥5 S/Co anti-HCV antibody-positive patients progressed with small increases until 2016; however, a significant downward trend was observed from 2017 onwards, with a significant annual decrease of around 4% during the entire study period (Figure 1). By contrast, the fluctuating number of antibody-positive patients within a narrow range was reflected as a significant and strong downward trend in the proportional unit, given that the number of tests increased by around 5% per year. This may be an indication that there is a certain pool of antibody-positive individuals in the population and that only a small number of new patients can be detected despite a significant increase in the number of tests. The decline in the number of positive patients, which has become more pronounced especially since 2020, together with the increase in the number of tests in the same period, has had a further strengthening effect on the proportional decline.

While vaccination is the most effective strategy for controlling viral diseases, unlike the hepatitis A and B viruses (HAV and HBV), no effective vaccine has yet been produced against HCV. The reason for this is the marked genetic diversity of the virus due to the high error rate of its RNA polymerase, resulting in the formation of antigenically distinct “quasispecies” populations even within the same host. The existence of at least eight genotypes and numerous subtypes has so far prevented the design of a vaccine that would provide widespread and durable protection due to the antigenic variability, which is particularly concentrated in envelope proteins [16]. This situation shows that HCV lacks an important prevention strategy to prevent community-wide spread, especially in pediatric infections, and makes other measures that can limit viral spread even more important.

Successful DAAs developed in recent years have made a significant contribution to the treatment of infected individuals while substantially reducing the infectiousness of patients. These drugs inhibit viral replication through three key targets—NS3/4A protease, NS5A and NS5B RNA polymerase—resulting in high viral suppression or cure rates (95%+), a 70% reduction in the risk of developing HCC and more than 85% reduction in liver-related mortality, and at least halving the spread of the virus in the community [17,18]. With a government communiqué published in May 2016, DAAs against HCV were included in the scope of general health insurance and patients in our country were provided access to these treatments at no cost. One of the most important results of our study was that the average number of viremic patients determined annually, which was around 175–180 in 2016 and before, decreased by 20% on average every year in 2017 and after (Table 2). In the proportional evaluation considering the entire study period, beta regression analysis revealed an annual decline of 12.5% (Figure 3). Despite the improved awareness in our country since the second half of the 1990s and the practices aimed at preventing the transmission of viral hepatitis since the beginning of the 2000s, our data showed that there was a significant viremic patient population in society until 2016 (Figure 3). However, after DAA treatments, this burden entered a very stable and strong downward trend and declined to approximately 10% of pre-DAA levels.

In our study, we analyzed age change over time for six different subpopulations. Regression analysis did not show any significant trend of change in the age characteristics of the population of patients who underwent anti-HCV antibody testing. This indicated that the test was generally used for population screening. The median age of positive patients was analyzed for three different patient subpopulations (all positives ≥1 S/Co, 1–4.99 S/Co and ≥5 S/Co) and a significant aging trend was found in high titer positives (≥5 S/Co) during the study period. In our study, a significant aging trend was also found in the patient population who underwent HCV-RNA testing and were found to be viremic. The median age analyses for patients indicated that, in our population as a whole, the inclusion of young or pediatric age groups in the HCV-infected population is low enough not to affect the overall trend, that the HCV-positive population infected at some point in their lives is steadily aging over time, and that after a few decades, the number of people living with HCV in our society will be significantly reduced. In individuals infected with HBV, another chronic hepatitis virus, a linear aging trend was found for a total of eight years over the entire 11-year study period, underlining the fact that this steady and efficient aging is based on the fact that pediatric or young adult patients are largely protected against the pathogen through routine vaccination [19]. These findings suggest that the current HCV-infected population will continue to age over time, and the overall disease burden is likely to decline in the coming decades due to the limited addition of new cases to the infected pool.

In our study, significant decreases were observed in the annual number of patients studied and the number of positive patients detected in 2020, and this situation could be attributed to the COVID-19 pandemic. Indeed, it has been reported that the COVID-19 pandemic has caused significant disruptions in the annual diagnosis and treatment processes of many chronic health problems or infectious diseases due to reasons such as prolonged curfews that started to be implemented with the onset of the pandemic, the understanding that the elderly have a higher risk of developing severe-fatal infections compared to other individuals in the population, and the obligation of health institutions to provide health care to pandemic patients in excess of their capacity [19,20]. In our study, it is understood that the diagnosis and treatment processes of HCV were also disrupted by the COVID-19 pandemic in 2020. Although not as much as the COVID-19 pandemic, a similar decrease in the volume of diagnoses was detected in 2023, and the reason for this situation was thought to be two consecutive massive earthquakes that occurred on February 6 and whose epicenter was very close to our city. Indeed, these earthquakes caused a major devastation 13 cities in our region, leaving hundreds of thousands of houses uninhabitable, tens of thousands of people dead and causing a significant human migration from our region. Therefore, our study demonstrated that HCV diagnostic processes are adversely affected in a pandemic affecting the whole world or a natural disaster affecting locally. Preparations are needed to ensure that HCV diagnosis and treatment processes are not adversely affected in such unexpected emergencies.

This study has several limitations. First, it is a retrospective single-center study based on laboratory records, which may limit the generalizability of the findings. Second, as the data were derived from hospital-based testing, the results reflect laboratory-based positivity trends rather than true population prevalence. Third, detailed clinical data, such as treatment status and disease stage, were not available. Finally, changes in testing practices and referral patterns over time may have influenced the observed trends.

## 5. Conclusions

In this study, it was found that anti-HCV positive patients in our region showed a decreasing trend over a period of 15 years, the number of viremic patients decreased markedly after the widespread use of DAA treatments in 2016, and positive patients turned into an older population over the years. In order to further reduce HCV infection and related chronic health problems and meet WHO targets, individuals who are unaware of their disease in the community should be effectively identified and included in the health care system and strict control of strengthened safe health practices should continue.

## Figures and Tables

**Table 1:** Characteristics of the patients tested for anti-HCV Ab from 2011 to 2025.

Characteristics	2011	2012	2013	2014	2015	2016	2017	2018	2019	2020	2021	2022	2023	2024	2025
**Total Test Number**	19,365	20,981	23,267	25,102	28,657	32,443	41,516	45,234	49,273	35,246	45,193	46,655	34,111	46,610	53,953
**Number of Tested Patient**	16,670	18,526	20,430	21,813	24,398	24,734	29,952	31,507	33,522	23,514	29,966	33,060	24,446	32,503	37,516
Median Age (year; min-max)	45; 0–107	43; 0–108	44; 0–113	42; 0–102	41; 0–107	39; 0–101	40; 0–102	40; 0–98	41; 0–100	41; 0–103	41; 0–103	42; 0–104	43; 0–100	42; 0–105	42; 0–101
Male	8878	9783	10,924	11,135	12,727	12,858	15,520	16,641	17,575	12,923	15,584	17,430	12,599	16,255	19,507
Female	7792	8743	9506	10,678	11,671	11,876	14,432	14,866	15,947	10,591	14,382	15,630	11,847	16,248	18,009
**Positive Patient** (≥1 S/Co; *n*)	417	468	519	521	553	543	585	605	613	389	456	507	317	410	407
Male	231	243	282	278	295	301	313	317	315	201	240	273	174	224	230
Female	186	225	237	243	258	242	272	288	298	188	216	234	143	186	177
Median Age (year; min-max)	56; 7–85	57; 0–89	58; 3–88	57; 0–97	58; 0–86	59; 2–89	58; 0–89	58; 1–92	58; 0–94	58; 0–87	58; 2–90	59; 1–96	61; 4–90	59; 0–93	59; 0–93
**Positive Patients** (≥1–4.99 S/Co; *n*)	158	171	168	195	194	177	229	275	271	187	239	261	160	213	234
Median Age (year; min-max)	46; 7–83	48; 0–89	50; 5–86	50; 0–90	47; 0–85	50; 2–89	47; 0–86	47.5; 1–92	47; 0–88	50; 0–86	49; 5–87	52; 1–95	56; 4–90	50; 0–88	53; 0–92
**Positive Patient** (≥5 S/Co; *n*)	259	296	351	326	359	366	356	330	342	202	217	246	157	197	173
Male	132	151	186	170	199	206	194	182	186	113	123	147	91	116	102
Female	127	145	165	156	160	160	162	148	156	89	94	99	66	81	71
Median Age (year; min-max)	60; 15–85	60; 16–84	60.5; 3–88	60; 18–97	60; 3–86	61; 6–88	61; 0–89	62; 9–89	62; 1–94	62.5; 5–87	63; 2–90	63; 3–96	63.5; 6–90	64; 21–93	64; 9–93
**New Patient** (≥5 S/Co; *n*)	163	143	181	149	144	130	116	101	105	62	78	84	42	61	51
**New Patient** (≥1–4.99 S/Co; *n*)	109	142	122	132	123	105	160	186	158	114	140	171	87	119	128

**Table 2:** Demographic characteristics and test results of patients who had HCV-RNA PCR during study period.

Year	Patients Tested				Patients with HCV Viremia		
	Test (*n*)	Patients (*n*)	Male/Female (*n*)	Median Age (Year; Min–Max)	Patient (*n*)	Male/Female (*n*)	Median Age (Year; Min–Max)
**2011**	848	596	322/274	53; 0–83	168	81/87	59; 15–83
**2012**	990	682	362/320	54; 0–90	162	80/82	60; 18–84
**2013**	892	665	342/323	56; 0–88	196	96/100	61; 18–85
**2014**	850	533	264/269	54; 0–87	161	77/84	61.5; 18–97
**2015**	837	570	304/266	53; 0–87	170	92/78	61.5; 19–86
**2016**	1101	734	394/340	56; 0–89	188	101/87	63; 8–88
**2017**	1064	702	383/319	55.5; 0–90	113	71/42	63; 9–86
**2018**	829	586	313/273	56; 1–92	76	46/30	64; 9–85
**2019**	902	685	373/312	57; 0–89	58	40/18	63.5; 6–80
**2020**	365	307	168/139	55.5; 0–87	22	12/10	56; 11–77
**2021**	788	512	289/223	57; 2–90	27	17/10	65; 21–83
**2022**	571	464	269/195	59; 1–96	27	18/9	64; 8–84
**2023**	436	381	222/159	56; 1–90	13	6/7	57.5; 26–79
**2024**	469	399	233/166	60; 0–86	27	22/5	67; 24–81
**2025**	387	278	156/122	60; 5–83	8	5/3	66; 20–83
**TOTAL**	**11,329**	**8094**	**4394/3700**		**1416**	**764/652**	

The temporal dynamics of the study population were evaluated using Poisson regression analysis. The annual trends in patient counts, revealed a significant, yet modest, decline in the total number of tested patients (*p* < 0.001). In contrast, a markedly more pronounced and statistically significant reduction was observed in the number of viremic (HCV-RNA positive) patients over the 15-year period (β = −0.178, *p* < 0.001), suggesting a substantial contraction of the active viral reservoir. Quantile regression (median) has indicated a consistent and significant aging trend within the general tested cohort, with the median age increasing by approximately 0.485 years annually (β = 0.485, *p* < 0.001).

## Data Availability

The data presented in this study are available from the corresponding author upon reasonable request. The data are not publicly available due to privacy and ethical restrictions, as they contain sensitive patient information obtained from hospital records.

## Abbreviations

The following abbreviations are used in this manuscript:

HAV	Hepatit A virus
HBV	Hepatit B virus
HCV	Hepatit C virus
DAAs	Direct-Acting Antivirals

## References

[1] World Health Organization (2024). Global Hepatitis Report 2024: Action for Access in Low- and Middle-Income Countries.

[2] U.S. Department of Health and Human Services (2020). Viral Hepatitis National Strategic Plan for the United States: A Roadmap to Elimination (2021–2025).

[3] Baecker A., Liu X., La Vecchia C., Zhang Z.F. (2018). Worldwide incidence of hepatocellular carcinoma cases attributable to major risk factors. Eur. J. Cancer Prev..

[4] Luo X., He Y., Jiang Z., Liao J. (2025). Global burden of liver cirrhosis: Trends from 1990–2021 and projections to 2060. J. Health Popul. Nutr..

[5] Săndulescu O., Dudman S.G., Gmizic I., Garcia F., Harvala H., Hasanoglu I., Janjua N.Z., Kowalska J., Mallet V., Maticic M. (2025). Global hepatitis C elimination: Updates, challenges, and opportunities from real-world experiences in Europe and North America. Hepatology.

[6] Republic of Türkiye Ministry of Health, General Directorate of Public Health (2025). Turkey Viral Hepatitis Control Program (2025–2030) (Ministry of Health Publication No. 1364).

[7] Panagiotakopoulos L., Sandul A.L., DHSc Conners E.E., Foster M.A., Nelson N.P., Wester C. (2023). CDC Recommendations for Hepatitis C Testing Among Perinatally Exposed Infants and Children—United States, 2023. MMWR Recomm. Rep..

[8] World Health Organization (2024). Hepatitis C.

[9] World Health Organization (2022). Global Health Sector Strategies on, Respectively, HIV, Viral Hepatitis and Sexually Transmitted Infections for the Period 2022–2030.

[10] Geddawy A., Ibrahim Y.F., Elbahie N.M., Ibrahim M.A. (2017). Direct Acting Anti-hepatitis C Virus Drugs: Clinical Pharmacology and Future Direction. J. Transl. Intern. Med..

[11] Idilman R., Razavi H., Robbins-Scott S., Akarca U.S., Örmeci N., Kaymakoglu S., Aygen B., Tozun N., Güner R., Bodur H. (2020). A micro-elimination approach to addressing hepatitis C in Turkey. BMC Health. Serv. Res..

[12] Republic of Türkiye Ministry of Interior, General Directorate of Security (2024). Turkey Drug Report 2024.

[13] Avcioglu F., Behcet M., Kalaycioglu O. (2021). Comparison of Anti HCV Signal-to-Cutoff Ratio with HCV RNA Results. Clin. Lab..

[14] Hussin A., Yusof R., Nor Rahim M.Y., Dalusim F., Othman N.I., Ibrahim N., Shahidan M.A. (2022). True-positive reflex threshold value for HCV antibody screening test. J. Infect. Dev. Ctries..

[15] Alter M.J., Kuhnert W.L., Finelli L. (2003). Guidelines for laboratory testing and result reporting of antibody to hepatitis C virus. Centers for Disease Control and Prevention. MMWR Recomm. Rep..

[16] Garbuglia A.R., Pauciullo S., Zulian V., Del Porto P. (2024). Update on Hepatitis C Vaccine: Results and Challenges. Viruses.

[17] Bang C.S., Song I.H. (2017). Impact of antiviral therapy on hepatocellular carcinoma and mortality in patients with chronic hepatitis C: Systematic review and meta-analysis. BMC Gastroenterol..

[18] Hajarizadeh B., Grebely J., Byrne M., Marks P., Amin J., McManus H., Butler T., Cunningham E.B., Vickerman P., Martin N.K. (2021). Evaluation of hepatitis C treatment-as-prevention within Australian prisons (SToP-C): A prospective cohort study. Lancet Gastroenterol. Hepatol..

[19] Tanriverdi E.S., Yakupogullari Y., Gunes M., Otlu B. (2026). A Decade of HBV DNA Testing: Trends in Positivity and Viral Load in a Tertiary Referral Laboratory in Türkiye. Viral Hepat. J..

[20] Yakupogullari Y., Ermis H., Kazgan Z., Otlu B., Bayindir Y., Gulbas G., Tanriverdi E., Guldogan E. (2022). Diagnostic and treatment outcomes of patients with pulmonary tuberculosis in the first year of COVID-19 pandemic. East. Mediterr. Health J..

